# Radiation Recall Pneumonitis: A Rare Syndrome That Should Be Recognized

**DOI:** 10.3390/cancers14194642

**Published:** 2022-09-24

**Authors:** Pei-Rung Jan, John Wen-Cheng Chang, Chiao-En Wu

**Affiliations:** 1 Department of Education, MacKay Memorial Hospital, Taipei 104, Taiwan; 2 Department of Traditional Chinese Medicine, Chang Gung Memorial Hospital at Linkou, Taoyuan 333, Taiwan; 3 Division of Hematology-Oncology, Department of Internal Medicine, Chang Gung Memorial Hospital at Linkou, Chang Gung University College of Medicine, 5 Fu-Hsing Street, Kwei-Shan, Taoyuan 333, Taiwan

**Keywords:** Radiation recall pneumonitis (RRP), radiation pneumonitis (RP), lung cancer, immunotherapy, targeted therapy, chemotherapy

## Abstract

**Simple Summary:**

A combination of radiotherapy and systemic antineoplastic agents is a common treatment strategy for lung cancer. However, Radiation recall pneumonitis (RRP) is a rare disease which has been mainly detected in the previously irradiated lung of patients with cancer after the application of triggering agents, including, but not limited to, antineoplastic agents. Physicians should be aware of this rare reaction, as the occurrence of RRP could impact the outcome of anti-cancer treatment. Given that current studies on RRP are primarily case reports and retrospectively reviewed data, the aim of our article was to review the current understanding and evidence on RRP and define the characteristics of RRP.

**Abstract:**

Radiation recall pneumonitis (RRP) is a rare but severe condition which has been mainly detected in the previously irradiated lung of patients with cancer after administering inciting agents, most commonly antineoplastic regimens including chemotherapy, targeted therapy, or immunotherapy. More recently, coronavirus disease vaccines were found to induce RRP. In addition to typical radiation pneumonitis (RP) or drug-induced interstitial lung disease, the management of RRP requires withholding inciting agents and steroid therapy. Thus, the occurrence of RRP could significantly impact cancer treatment, given that inciting agents are withheld temporarily and even discontinued permanently. In the present review, we discuss the current understanding and evidence on RRP and provide additional insights into this rare but severe disease.

## 1. Introduction

**Figure 1:**
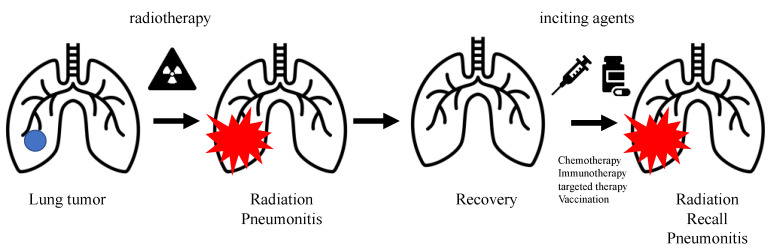
Radiation pneumonitis (RP) commonly occurs in patients treated with radiotherapy to the lung. After recovery from RP, radiation recall pneumonitis (RRP) may occur in the previously irradiated lung of patients with cancer after administering inciting agents, including chemotherapy, targeted therapy, immunotherapy, and vaccination.

Radiation recall is an acute inflammatory reaction of a previously irradiated field after the application of triggering agents, including, but not limited to, antineoplastic agents [1], anti-tuberculosis drugs [2], antibiotics [3], tamoxifen [4], and simvastatin [5]. Radiation recall reactions may occur in the mucosa, lungs, muscles, and gastrointestinal tract [1]. Radiation recall dermatitis (RRD) has been widely reported, whereas Radiation recall pneumonitis (RRP) remains a rare but more severe syndrome primarily observed in the previously irradiated lung of patients after administering systemic antineoplastic regimens. More recently, in addition to antineoplastic agents such as chemotherapy [1,6,7,8,9,10], immunotherapy [11,12,13,14,15,16,17,18,19,20,21,22,23], and targeted therapy [11,24,25,26,27,28,29,30,31,32], vaccination [33,34,35] has been associated with RRP (Figure 1).

**Figure 2:**
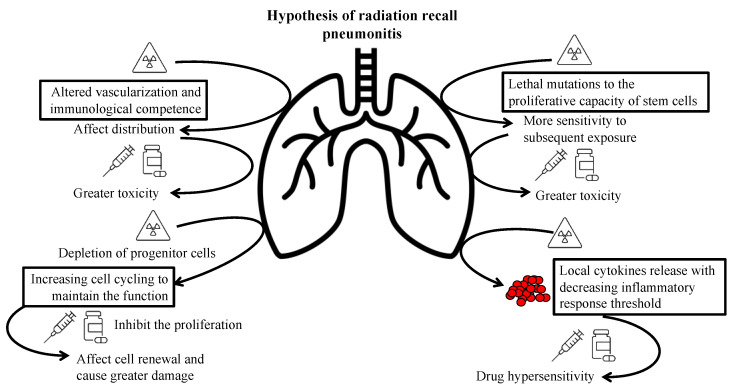
Summary of possible hypotheses of radiation recall pneumonitis.

The precise pathophysiological mechanism of RRP remains unclear; however, several hypotheses have been proposed. One hypothesis postulates that radiotherapy alters vascularization and the immunological competence of fibrotic tissue, potentially impacting the distribution of the inciting drugs, resulting in greater toxicity in previously irradiated areas than in unirradiated areas [36]. According to another hypothesis, radiotherapy induces lethal mutations in the proliferative capacity of stem cells, which consequently causes the previously irradiated but apparently healed tissue to demonstrate remarkable sensitivity to subsequent irradiation or cytotoxic drug exposure [37]. Examining observations and analysis of RRD, persistent depletion of progenitor cells and increased cell cycling have been documented in the irradiated epidermis to maintain function [5]. Thus, agents that inhibit the proliferation impact cell renewal in the injured tissue and facilitate damage induction. However, these hypotheses fail to clarify the randomness of the radiation recall effect and why non-cytotoxic drugs cause radiation recall. Another hypothesis of drug hypersensitivity reactions meets the characteristics of RRP [16]. Although radiation induces the release of cytokines [20], such as interleukin-1, interleukin-6, and tumor necrosis factor-alpha, certain drugs could trigger a non-immune inflammatory reaction in patients with an irradiation-induced reduction in the inflammatory response threshold [38]. COVID-19 vaccine-induced RRP may share similar mechanisms as above, since the inflammatory state created by the vaccine could also trigger a hypersensitivity reaction in the previously irradiated area [33]. Figure 2 summarizes the potential hypotheses.

Moreover, the incidence, characteristics, and distinction between RRP and typical radiation pneumonitis (RP) or drug-induced pneumonitis are poorly defined. Thus, in the present review article, we discuss the current understanding and evidence on RRP, which may provide further insights into such rare diseases.

## 2. Chemotherapy-Induced RRP

A combination of radiotherapy and chemotherapy is a common treatment strategy for lung cancer. Meanwhile, several chemotherapeutic regimens, including taxanes [8], anthracyclines [6,7], and gemcitabine [9], were found to be associated with RRP. For example, in the 20th century, McLnerney and Bullimore [6] presented a 4-year-old girl with metastatic nephroblastoma treated with thoracic radiotherapy and concurrent chemotherapy with vincristine, actinomycin D, and adriamycin, who experienced RP three months after the completion of radiotherapy. Four months after radiotherapy, she developed dyspnea one day after a planned adriamycin dose, and chest radiography revealed extensive opacification in the previous RP area. Her symptoms gradually resolved with prednisolone, while no adverse effects were noted during the subsequent actinomycin D and adriamycin administration with concurrent prednisolone 5 mg/day. Schweitzer et al. presented a 61-year-old female receiving thoracic radiation of 46 Gy for lung adenocarcinoma that metastasized to the right ribs. The patient developed skin erythema and shortness of breath several hours after paclitaxel infusion on day 12 after radiotherapy completion. The patient presented with a prolonged dry cough, dyspnea, and parenchymal opacity corresponding to the radiation portal on a follow-up chest radiograph performed three weeks later. Five weeks after the occurrence of RRP, the patient tolerated the second dose of paclitaxel well, without exhibiting pulmonary symptoms, under premedication with dexamethasone (20 mg), 24, 12 h, and immediately before paclitaxel administration. The following week, the chest radiograph showed an improvement in parenchymal opacity.

Azria et al. [1] searched the Medline and CancerLit databases to identify reports on the radiation recall phenomenon and revealed that taxanes and anthracyclines were responsible for 20% and 30% of radiation recall reactions, respectively.

Ding et al. [10] presented a case series of 12 patients with lung cancer diagnosed with RRP induced by chemotherapeutic regimens, including taxanes, gemcitabine, etoposide, vinorelbine, and epirubicin. The median radiation dose was 60.7 Gy (range, 52–66 Gy). The median time interval between radiotherapy completion and RRP, and between chemotherapy initiation and RRP, was 95 days (range, 71–202 days) and 47 days (range, 22–169 days), respectively. Of these 12 patients with RRP, 7 underwent a chemotherapy rechallenge, with 3 rechallenged with the same agents and 1 with the same kind of agents. It should be noted that rechallenged patients showed no recurrence with concurrent steroid use. Accordingly, patients could be successfully rechallenged with efficient agents and concurrent steroid use despite the previous occurrence of RRP.

The clinical courses of previous reports of chemotherapy-induced RRP are summarized in Table 1.

## 3. Immunotherapy-Induced RRP

In recent years, the blockade of programmed death 1 (PD-1) and programmed death ligand 1 (PD-L1) has been used to treat advanced non-small cell lung cancer (NSCLC). For example, pembrolizumab was found to exhibit longer progression-free and overall survival than platinum-based chemotherapy in treatment-naive patients with advanced NSCLC with PD-L1 expression ≥50% [12]. Nivolumab afforded better overall survival, response rate, and progression-free survival than docetaxel in patients with advanced, previously treated squamous cell and non-squamous cell NSCLC, regardless of PD-L1 expression levels [39,40]. In addition, durvalumab demonstrated longer recurrence-free survival than the placebo in patients with stage III advanced NSCLC without disease progression after concurrent chemoradiotherapy [13]. However, an overlapping effect of immunotherapy and radiotherapy on pulmonary toxicity was observed in patients with NSCLC, particularly in those undergoing concurrent radiotherapy and immunotherapy.

In a phase 1 KEYNOTE-001 trial [14], patients who previously received thoracic radiotherapy were more likely to experience pulmonary toxicity, especially pneumonitis, after the application of pembrolizumab. In a phase 2 PEMBRO-RT trial [17], pneumonitis occurred more frequently in patients who received pembrolizumab combined with radiotherapy than in those who did not receive radiotherapy (33.9% vs. 24.8%). McGovern et al. [16] presented a case of an 82-year-old male patient receiving pembrolizumab monotherapy four months after the completion of radiotherapy and who subsequently developed asymptomatic fluorodeoxyglucose (FDG)-avid right upper lung infiltration in a previously irradiated field 10 months after initiating pembrolizumab therapy. Itamura et al. [19] also presented a case of asymptomatic, typical RP, who then experienced severe symptomatic RRP with shortness of breath on exertion and decreased oxygen saturation 35 days after pembrolizumab administration and 6 months after the radiotherapy completion.

Shibaki et al. [15] reported two patients who received thoracic radiotherapy two years earlier and developed symptomatic pneumonitis, along with fever, dyspnea, and decreased oxygen saturation six weeks and six months after nivolumab administration. Both computed tomography (CT) images showed opacities matching the irradiated field; therefore, RRP was suspected. The symptoms promptly improved after four weeks of oral prednisolone therapy.

In a phase 3 PACIFIC trial [13], patients who received durvalumab had a higher incidence of pneumonitis or RP than those who received a placebo (any grades: 33.9% and 24.8%, grade 3 or more: 3.4% and 2.6%, respectively). In addition, Wang et al. [23] reported a 54-year-old female patient who received concurrent chemoradiotherapy at a dose of 60 Gy for locally advanced NSCLC. The patient then experienced a cough and dyspnea, which was diagnosed as RRP during the tenth cycle of sintilimab, 10 months after completing radiotherapy. The symptoms gradually resolved after discontinuing sintilimab and 4-week prednisolone therapy. Chen et al. [18] presented a case of a 64-year-old male patient with NSCLC who received chemoradiotherapy with cisplatin and pemetrexed for simultaneous integrated boost radiotherapy and started a PD-1 blockade with camrelizumab owing to tumor progression. The patient subsequently developed dyspnea and a cough after the eighth camrelizumab administration, and the chest CT revealed patchy consolidation and ground–glass opacities localized within the previously irradiated area. The symptoms and CT images improved within two weeks after camrelizumab cessation and treatment with prednisolone.

Cousin et al. [21] reviewed the medical records and CT images of patients treated with PD-1 or PD-L1 inhibitors, including pembrolizumab, nivolumab, and atezolizumab, for advanced lung cancer. The authors revealed that the incidence of RRP was 18.8% (15 out of 80 patients) without any identified risk factors. Among these patients, the median time between radiotherapy completion and RRP was 450 days (range, 231–1859 days), while the median time between immunotherapy initiation and the occurrence of RRP was 61 days (range, 4–520 days). However, only 5 of the 15 (33.3%) patients experienced symptomatic RRP. RRP did not impact treatment outcome, given that no significant difference in progression-free survival was noted between patients with or without RRP.

As monotherapy with immune checkpoint inhibitor can induce RRP, it is unsurprising that combining dual checkpoint inhibitors or checkpoint inhibitors with targeted therapy can induce RRP. Riviere et al. [22] presented three cases of RRP after initiating a nivolumab experimental histone deacetylase inhibitor, ipilimumab–pembrolizumab, and nivolumab–ipilimumab, 4.5 years, ~6 months, and 7 months after radiotherapy completion, respectively.

It is worth noting that immune checkpoint inhibitors commonly induce interstitial lung disease; therefore, differentiating RRP from typical drug-induced pneumonitis can be challenging without a CT scan to identify pneumonitis in previously irradiated fields. In addition, the reported intervals from radiation or immune checkpoint inhibitors to the occurrence of RRP varied from months to years. Thus, RRP should always be considered once patients with previous thoracic irradiation develop pneumonitis.

Table 2 summarizes the clinical courses of previous reports on immunotherapy-induced RRP.

## 4. Targeted Therapy-Induced RRP

With the evolution of genetic testing and antineoplastic treatment, a growing number of targeted therapies have been developed, accompanied by an increasing number of targeted therapy-induced RRPs.

### 4.1. Epithelial Growth Factor Receptor (EGFR)–Tyrosine Kinase Inhibitors (TKIs)

EGFR-TKIs are the most widely employed targeted therapies for advanced lung cancer harboring EGFR mutations, and EGFR-TKI-induced RRP has been frequently documented. Awad et al. [30] reported a 76-year-old male patient with NSCLC who was treated with pemetrexed as maintenance chemotherapy. Owing to disease progression, he received palliative thoracic radiotherapy of 30 Gy, and erlotinib nine weeks after completing radiotherapy. Thereafter, he experienced severe dyspnea, cough, reduced oxygen saturation, anorexia, and fatigue. Chest CT showed air space opacification over the bilateral perihilar areas, consisting of an irradiated field two months after erlotinib administration and three months after completing radiotherapy. Erlotinib was discontinued, and the symptoms were relieved rapidly following prednisolone therapy. Four weeks later, a repeat CT scan revealed the resolution of the pneumonitis. Nevertheless, his respiratory symptoms remained stable after a rechallenge with erlotinib 11 weeks later.

Chiang et al. [31] retrospectively reviewed the clinical records and consecutive chest images of 160 patients who received EGFR-TKIs after thoracic radiotherapy. Therapy included gefitinib and erlotinib, while the median radiation dose was 60 Gy (range, 20–76 Gy), and the median interval between the completion of radiotherapy and EGFR-TKI was 7.4 months (range, 0.2–55 months). Among these patients, acute interstitial pneumonitis developed in 20 patients (12.5%) and EGFR-TKI-induced RRP was documented in 7 patients (4.4%). The key point in distinguishing RRP from typical EGFR-TKI-induced interstitial lung disease is that RRP is confined to the prior radiation field, instead of exhibiting bilateral and random distribution. The median time interval between radiotherapy completion and RRP was 124 days (range, 80–635 days), whereas that between EGFR-TKI initiation and RRP was 43 days (range, 18–65 days). Notably, patients who initiated EGFR-TKI therapy within 90 days of radiotherapy completion exhibited significantly higher rates of RRP than those who initiated EGFR-TKI treatment after 90 days (21% vs. 2.1%, *p* = 0.005).

In addition to gefitinib and erlotinib, cases of osimertinib-induced RRP have been reported. Sanchis–Borja et al. [32] presented a 58-year-old male patient with locally advanced lung adenocarcinoma who was treated with first-line cisplatin and gemcitabine chemotherapy, affording partial tumor response, followed by gefitinib for EGFR exon 21 L858R mutation. However, local lung progression with T790M resistance mutation was noted; thus, the patient underwent osimertinib and carboplatin–paclitaxel doublet with bevacizumab. Following the newly discovered local progression, thoracic radiotherapy of 55 Gy was performed. Osimertinib was withheld seven days before radiotherapy and resumed two months later. Two weeks later, he was hospitalized for dyspnea and fever, with a CT scan revealing peri-bronchial consolidation and ground–glass opacities predominating in the irradiated field. Bronchoalveolar lavage (BAL) of the right lower lobe revealed major lymphocytic alveolitis. His symptoms and pulmonary consolidation regressed after osimertinib discontinuation and prednisolone therapy.

### 4.2. Vascular Endothelial Growth Factor (VEGF) Receptors

Sunitinib is a small-molecule TKI that primarily inhibits tumor angiogenesis by blocking targets, including VEGF receptors, and is commonly used to treat gastrointestinal stromal tumor (GIST) and metastatic renal cell carcinoma (mRCC). Seidel et al. [26] presented a case report of a 49-year-old female patient with metastatic clear cell renal carcinoma who received palliative radiotherapy of 30 Gy. Six months after completing radiotherapy, the patient developed a cough during the fourth course of sunitinib. A CT scan revealed a new ground–glass opacity within the previously irradiated area, and a BAL revealed that the lymphocytes increased to 14%. Her symptoms resolved three weeks after reducing the dosage of sunitinib without steroid coverage; however, the interstitial changes remained detectable in follow-up CT images.

### 4.3. Mammalian Target of Rapamycin (mTOR) Inhibitors

Everolimus is a protein kinase inhibitor of the mTOR serine/threonine kinase signal transduction pathway, which regulates cell growth, proliferation, and survival, and is frequently deregulated in cancer [41]. Motzer et al. [24] evaluated the efficacy of everolimus in patients with mRCC who had failed prior targeted therapy with sunitinib or sorafenib; the everolimus-treated patients showed longer progression-free survival than the placebo group (4.0 vs. 1.9 months). Therefore, everolimus is widely employed as later-line therapy for mRCC [42]. Clark et al. [27] reported a 58-year-old female patient with recurrent mRCC in the left lower lung who received palliative radiotherapy of 39 Gy and initiated everolimus one month later. She subsequently experienced a dry cough, shortness of breath with hypoxemia, low-grade fever, nausea, and vomiting one month after everolimus initiation. CT imaging revealed a new patchy consolidation with confluent ground–glass opacities. Everolimus was suspended. During hospitalization, supportive care with high-flow oxygen and steroid therapy was provided, and her symptoms gradually improved six weeks later.

### 4.4. Human Epithelial Growth Factor-2 (HER-2) Inhibitors

Trastuzumab is a recombinant monoclonal antibody against HER-2. One year of adjuvant trastuzumab after chemotherapy was found to significantly improve overall survival and disease-free survival in females with HER2-positive early breast cancer, as determined in a long-term follow-up of the HERA trial [43]. In previous reports, trastuzumab induced dermatitis as a radiation recall reaction [11,25]. Lee et al. [29] presented a 55-year-old female patient with a right breast fibroadenoma who received radiotherapy of 50.4 Gy after surgery. During the eighth course of trastuzumab, two years after completing radiotherapy, she consecutively developed erythematous and edematous plaque on the right breast and dyspnea, accompanied by a dry cough and parenchymal opacity corresponding to the radiation dose distribution on the CT scan. RRD and RRP were diagnosed simultaneously, and her symptoms improved after two-week prednisolone therapy.

### 4.5. BRAF Inhibitors

In patients with unresectable metastatic melanoma harboring a BRAF V600E mutation, vemurafenib, a BRAF inhibitor, afforded a higher response rate and longer progression-free survival and overall survival than chemotherapy with dacarbazine [44]. Forschner et al. [28] presented two cases of metastatic melanoma with a BRAF V600E mutation that developed RRP after vemurafenib treatment. One patient was a 71-year-old male receiving vemurafenib four weeks after completing well-tolerated radiotherapy of 50 Gy. The patient experienced a dry and persistent cough, with a newly detected ground–glass appearance in the irradiated field after three weeks of vemurafenib therapy. The second patient was a 47-year-old female with a history of RP who received vemurafenib three weeks after completing radiotherapy at 40 Gy and developed heavy breathing; the thoracic CT revealed ground–glass opacity and consolidation in the irradiated para-mediastinal region. Both symptoms improved promptly within 10 days of prednisolone therapy with prophylactic antibiotics, particularly without discontinuing vemurafenib.

Table 3 summarizes the clinical courses of previous reports on targeted therapy-induced RRP.

## 5. Vaccination-Induced RRP

During the coronavirus disease 2019 (COVID-19) pandemic, mRNA vaccines were found to be a common and efficient choice. However, the side effects and drug interaction of mRNA vaccines remain ambiguous. Three cases of potential COVID-19 vaccination-induced RRP have been documented.

First, Steber et al. [33] presented a 66-year-old male patient receiving local consolidation radiotherapy and chemoimmunotherapy for oligometastatic NSCLC, who developed pneumonitis within 3 days of receiving his first dose of the Moderna COVID-19 vaccine, which progressed after administering the second vaccine dose one month later. Shinada et al. [35] presented a 48-year-old male subject receiving chemoradiotherapy for locally advanced NSCLC, who developed a fever and dry cough 19 days after the second dose of the BNT162b2 COVID-19 vaccine. The patient exhibited an infiltration shadow in an area overlapping the previous radiation field on CT images. Both patients recovered quickly after prednisolone administration. Finally, Hughes et al. [34] presented serial fluorodeoxyglucose (FDG)–positron emission tomography (PET)-CT images of a 67-year-old male patient receiving thoracic radiotherapy for residual lung adenocarcinoma who developed pneumonitis three months after the second mRNA COVID-19 vaccination dose. Symptoms resolved simultaneously without any intervention.

Table 4 summarizes the clinical courses of previous reports of vaccination-induced RRP.

## 6. Discussion

In summary, RRP is diagnosed based on a history of previous irradiation and the application of systemic agents when compared with RP. The occurrence of RRP is unpredictable and occurs months to years after treatment. Previously reported cases experienced pulmonary symptoms that could be relieved with a short course of steroids. Age, sex, dosage and program of radiotherapy, history of smoking, previous RP, or interstitial lung disease do not indicate an increased prevalence of RRP based on previous case series [21]. Moreover, there is no obvious association between the occurrence of RRP and the time intervals from radiotherapy completion, or the initiation of systemic agents and the occurrence of RRP.

Typical symptoms of RRP include a dry or productive cough, shortness of breath with or without hypoxemia, chest tightness, chest pain, and low-grade fever. The course of RRP lasts days to weeks. The classic radiologic manifestations of RRP include ground–glass opacity, diffuse infiltration, or patchy consolidation, which corresponds to the shape and size of the radiotherapeutic program. In addition, BAL may reveal an increased lymphocyte count. To the best of our knowledge, there is no obvious dissimilarity in RRP induced by distinctly categorized agents.

To distinguish the severity of RRP, we referred to the grading system of pneumonitis in the Common Terminology Criteria for Adverse Effects. As RRP is unpredictable and rarely identified, symptoms categorized as ≥ grade 2 must be carefully monitored and may require oxygen support or medical intervention. Moreover, it is important to distinguish RRP from typical RP and drug-induced pneumonitis, given that disrupting or altering antineoplastic regimens would significantly affect cancer treatment outcomes, and progressive pneumonitis may be fatal to patients. To the best of our knowledge, RRP typically resolves faster than RP following steroid therapy and seldom induces persistent lung fibrosis and scarring.

As mentioned earlier, withholding inciting agents and steroid therapy play major roles in managing RRP. No standard steroid dosage has been established. Typically, treatment with prednisolone 0.5–2 mg/kg/day should be undertaken, followed by titration based on symptom severity. Furthermore, rechallenges with the same regimens can pose a crucial issue for patients with cancer experiencing RRP. In some cases, rechallenge therapy with the same regimens did not induce RRP recurrence under steroid coverage, indicating the possibility of maintaining efficient treatment regimens despite a history of RRP.

A history of RP was not necessary for the diagnosis of RRP. Previous studies have shown that some patients with RRP had no previous RP occurrence. This may be attributed to mild injury to the previously irradiated lung, leading to no obvious radiological abnormality and asymptomatic RP (grade 1) without a radiological diagnosis. Unless regular follow-up of radiological images is performed, such as chest radiography or high-resolution CT (HRCT), some patients with RP may be overlooked. Therefore, it can be difficult to differentiate RRP from RP if pneumonitis occurs after completing radiotherapy and initiating systemic treatment for those without a previous history of RP. However, the treatment strategy should be the same for both RP and RRP. In addition, it is challenging to identify the inciting agent(s) when patients are treated with multiple drugs (in combination with immunotherapy, chemotherapy, targeted therapy) and vaccinations simultaneous to RRP occurrence.

The radiation program and racial differences may influence the occurrence of RRP. Therefore, we included the details of irradiation techniques, total radiation dose, number of fractions, and mean lung dosage. In contrast, race was difficult to be identified in previous case reports. Publication bias may exist for further analysis so we should not analyze the impact of the radiation program and racial differences on RRP.

## 7. Conclusions

In conclusion, RRP is an acute inflammatory reaction of a previously irradiated lung after the application of triggering agents. Apart from typical RP and drug-induced pneumonitis, RRP is diagnosed base on a history of irradiation and the application of systemic agents. According to previous reports of RRP, antineoplastic regimens including chemotherapy, targeted therapy, and immune-therapy are common inciting agents. During the pandemic, coronavirus disease vaccines were also found to induce RRP. It is important to recognize the occurrence of RRP since the management includes withholding the inciting agents as well as a concurrent steroid therapy.

Given that current studies on RRP are primarily case reports and retrospectively reviewed data, further research is needed to accurately define the characteristics of RRP. Physicians should be aware of this rare reaction, which may affect the decisions and outcomes of anti-cancer treatment.

## Figures and Tables

**Table 1:** Summary of published cases of RRP induced by chemotherapy.

Drugs (Cases ^∑^)	Radiotherapy Program ^#^	Time Interval from Radiotherapy *	Time to Onset ^&^	Treatment ^∆^	Rechallenge with Same Regimen
Adriamycin [6]	Co-60 radiation/ 15 Gy/10 F/ unknown	4 months	1 day	Prednisolone 20 mg/day, gradually tapered to 5 mg/day across 3 weeks	No recurrence with concurrent prednisolone 5 mg/day
Paclitaxel [8]	Palliative RT/ 43.2 Gy/24 F/ unknown	12 days	Several hours	Dexamethasone 20 mg once	No recurrence with premedication with 3 doses of dexamethasone 20 mg
Cyclophosphamide, epirubicin, and vincristine [10]	3D-CRT/ 60 Gy/30 F/ 15.6 Gy	71 days	29 days	Systemic steroids, dose not reported	No
Docetaxel [10]	IMRT/ 54 Gy/24 F/ 14.89Gy	82 days	51 days		No
Gemcitabine and docetaxel (*n* = 2) [10]	3D-CRT/ 62 Gy/34 F/ 14.46 Gy	87.5 days (range, 81–94 days)	30 days (range, 22–38 days)		No recurrence with concurrent steroid coverage
Carboplatin and Etoposide [10]	IMRT/ 60 Gy/30 F 13.19 Gy	94 days	79 days		No
Navelbine and cisplatin [10]	3D-CRT/ 52 Gy/26 F 18.19 Gy	102 days	42 days		No
Paclitaxel and carboplatin (*n* = 5) [10]	3D-CRT (*n* = 4) and IMRT (*n* = 1)/ 62.52 Gy/33.2 F/ 17.036 Gy	105 days (range, 86–202 days)	71 days (range, 36–169 days)		1 of 5 patients was rechallenged and no recurrence with concurrent steroid coverage was found
Etoposide and cisplatin [10]	IMRT/ 60 Gy/30 F/ 14.44 Gy	171 days	164 days		No

3D-CRT = Three-dimensional conformal RT; IMRT = intensity-modulated RT. ^#^ Details of radiotherapy program, presented with irradiation techniques/total radiation dose (Gy)/number of fractions (F)/mean lung dosage (Gy). In the cases with more than one patient, average numbers were presented. * Time interval for radiotherapy = time interval between the onset of RRP and the radiotherapy completion. ^&^ Time to onset = time interval between the onset of RRP and the inciting agents initiation. ^∆^ Treatment included withholding the inciting agents, except for the marked management. ^∑^ The case number only presented the case of more than one patient.

**Table 2:** Summary of published cases of RRP induced by immunotherapy.

Drugs (Cases ^∑^)	Radiotherapy Program ^#^	Time Interval from Radiotherapy *	Time to Onset ^&^	Treatment ^∆^	Rechallenge with Same Regimen
Pembrolizumab [16,19]	EBRT/ unknown/unknown/ 5–20 Gy	14 months	10 months	Prednisolone 1 mg/kg, followed by a prolonged taper for 3 months	Not reported
	3D-CRT/ 64 Gy/32 F/ 15 Gy	7 months	1 month	Methylprednisolone 1000 mg/day for 3 days, then prednisolone 60 mg/day tapered in 3 months	Not reported
Nivolumab [15]	Unknown/ 60 Gy/unknown/ unknown	2 years	6 weeks	Prednisolone 1 mg/kg, tapered over 4 weeks	Not reported
	Unknown/ 60 Gy/unknown/ unknown	2 years	6 months	Prednisolone 1 mg/kg, tapered over 4 weeks	Not reported
Sintilimab [23]	CCRT/ 60 Gy/30 F/ 13.5 Gy	11 months	10 months	Prednisolone 120 mg twice a day, tapered over 4 weeks	Not reported
Camrelizumab [18]	SIB/ 63.8 Gy/29 F/ 13.5 Gy	19 months	4 months	Prednisolone 80 mg twice a day, tapered over 3 weeks	Not reported
Pembrolizumab Nivolumab Atezolizumab (*n* = 15) [21]	CRT (*n* = 12) and SBRT (*n* = 3)/ 60 Gy/unknown/ 11 Gy	450 days (range, 231–1859 days)	61 days (range, 4–520 days)	Not reported	Not reported
Nivolumab and experimental histone deacetylase inhibitor [22]	IMRT/ 59.4 Gy/33 F/ Unknown	4.5 years	2 weeks	Prednisolone 60 mg/day, tapered gradually	Not reported
Ipilimumab and pembrolizumab [22]	SBRT/ 25 Gy/5 F/ unknown	Less than half a year	3 days (second dose)	Expired	Expired
Nivolumab and Ipilimumab [22]	CRT/ 30 Gy/10 F/ unknown	7 months	11 days (fourth dose)	Prednisolone 50 mg/day, tapered gradually	Not reported

EBRT = external beam RT; CCRT = concurrent chemoradiotherapy; SIB = simultaneous-integrated boost RT; CRT = conventional RT. ^#^ Details of radiotherapy program, presented with irradiation techniques/total radiation dose (Gy)/number of fractions (F)/mean lung dosage (Gy). In the cases with more than one patient, average numbers were presented. * Time interval for radiotherapy = time interval between the onset of RRP and the radiotherapy completion. ^&^ Time to onset = time interval between the onset of RRP and the inciting agents initiation. ^∆^ Treatment included withholding the inciting agents, except for the marked management. ^∑^ The case number only presented the case of more than one patient.

**Table 3:** Summary of published cases of RRP induced by targeted therapy.

Drugs (Cases ^∑^)	Radiotherapy Program ^#^	Time Interval from Radiotherapy *	Time to Onset ^&^	Treatment ^∆^	Rechallenge with Same Regimen
Erlotinib [30]	Palliative/ 30 Gy/12 F/ 10.7Gy	4 months	2 months	Prednisolone 50 mg/day, tapered over 4 weeks	No recurrence without mention of steroid coverage
Gefitinib and erlotinib (*n* = 7) [31]	Conventional and conformal RT/ 60 Gy/unknown/ 12.8 Gy	124 days (range, 80–635 days)	43 days (range, 18–65 days)	Systemic steroid for grade 3 RRP (*n* = 3)	No recurrence in patients with grade 1 and 2 RRP without mention of steroid coverage One patient with grade 3 RRP developed interstitial pneumonitis after rechallenge
Osimertinib [32]	Hypo-RT/ 55 Gy/20 F/ unknown	2.5 months	2 weeks (pre-exposure)	Prednisolone 0.5 mg/kg/day for 1 week	Not reported
Sunitinib [26]	Palliative RT/ 30 Gy/unknown/ unknown	6 months	5 months	Reduced the sunitinib dose from 50 to 37.5 mg/day	Dose adjustment, no discontinuation of sunitinib
Everolimus [27]	Palliative RT/ 39 Gy/13 F/ unknown	2 months	1 month	Methylprednisolone followed by oral prednisolone, not reported dose	Not reported
Trastuzumab [29]	Unknown/ 50.4 Gy/unknown/ unknown	2 years	2 years	Prednisolone 30 mg/day for 2 weeks	Not reported
Vemurafenib [28]	3D-CRT/ 50 Gy/25 F 6.9 Gy	7 weeks	3 weeks	Continued vemurafenib ^∆^ Added prednisolone 150 mg/day for 10 days	No discontinuation of vemurafenib
	3D-CRT/ 50 Gy/25 F/ 17.4 Gy	7 weeks	4 weeks	Continued vemurafenib ^∆^ Added prednisolone 60 mg/day for 10 days	No discontinuation of vemurafenib

Hypo-RT = Hypofractionated RT. ^#^ Details of radiotherapy program, presented with irradiation techniques/total radiation dose (Gy)/number of fractions (F)/mean lung dosage (Gy). In the cases with more than one patient, average numbers were presented. * Time interval for radiotherapy = time interval between the onset of RRP and the radiotherapy completion. ^&^ Time to onset = time interval between the onset of RRP and the inciting agents initiation. ^∆^ Treatment included withholding the inciting agents, except for the marked management. ^∑^ The case number only presented the case of more than one patient.

**Table 4:** Summary of published cases of RRP induced by vaccination.

Drugs (Cases ^∑^)	Radiotherapy Program ^#^	Time interval from Radiotherapy *	Time to Onset ^&^	Treatment ^∆^	Rechallenge with Same Regimen
Moderna COVID-19 vaccine [33]	Unknown/ 45 Gy/15 F/ unknown	6 months	3 days (second dose)	Prednisolone 40 mg/day and tapered gradually	Not reported
BNT162b2 COVID-19 vaccine [35]	IMRT/ 60 Gy/30 F/ unknown	1 year	19 days (second dose)	Prednisolone 0.5 mg/kg/day and tapered gradually	Not reported
mRNA COVID-19 vaccine [34]	Unknown/ 60 Gy/15 F/ unknown	8 months	3 months (second dose)	No intervention ^∆^	Not reported

^#^ Details of radiotherapy program, presented with irradiation techniques/total radiation dose (Gy)/number of fractions (F)/mean lung dosage (Gy). In the cases with more than one patient, average numbers were presented. * Time interval for radiotherapy = time interval between the onset of RRP and the radiotherapy completion. ^&^ Time to onset = time interval between the onset of RRP and the inciting agents initiation. ^∆^ Treatment included withholding the inciting agents, except for the marked management. ^∑^ The case number only presented the case of more than one patient.
